# The Multifaceted Role of Plasminogen in Cancer

**DOI:** 10.3390/ijms22052304

**Published:** 2021-02-25

**Authors:** Beate Heissig, Yousef Salama, Taro Osada, Ko Okumura, Koichi Hattori

**Affiliations:** 1 Immunological Diagnosis, Juntendo University, School of Medicine, 2-1-1 Hongo, Bunkyo-Ku, Tokyo 113-8421, Japan; kokumura@juntendo.ac.jp; 2 An-Najah Center for Cancer and Stem Cell Research, Faculty of Medicine and Health Sciences, An-Najah National University, Nablus P.O. Box 7, Palestine; yousef.ut@najah.edu; 3 Department of Gastroenterology Juntendo University Urayasu Hospital, 2-1-1 Tomioka, Urayasu-shi, Chiba 279-0021, Japan; otaro@juntendo.ac.jp; 4 Center for Genomic & Regenerative Medicine, Juntendo University, School of Medicine, 2-1-1 Hongo, Bunkyo-Ku, Tokyo 113-8421, Japan; khattori@ims.u-tokyo.ac.jp

**Keywords:** cancer, plasminogen, metastasis, drug resistance, exosomes, uPAR, senescence, LRP1, anoikis, premetastatic niche

## Abstract

Fibrinolytic factors like plasminogen, tissue-type plasminogen activator (tPA), and urokinase plasminogen activator (uPA) dissolve clots. Though mere extracellular-matrix-degrading enzymes, fibrinolytic factors interfere with many processes during primary cancer growth and metastasis. Their many receptors give them access to cellular functions that tumor cells have widely exploited to promote tumor cell survival, growth, and metastatic abilities. They give cancer cells tools to ensure their own survival by interfering with the signaling pathways involved in senescence, anoikis, and autophagy. They can also directly promote primary tumor growth and metastasis, and endow tumor cells with mechanisms to evade myelosuppression, thus acquiring drug resistance. In this review, recent studies on the role fibrinolytic factors play in metastasis and controlling cell-death-associated processes are presented, along with studies that describe how cancer cells have exploited plasminogen receptors to escape myelosuppression.

## 1. Introduction

Proteolysis is required during normal development, as well as in pathological conditions, such as cancer and inflammation. Enzymes of the fibrinolytic cascade and their inhibitors contribute to carcinogenesis, cancer growth progression, and metastasis through a fine-tuned activation and deactivation of the contributing enzymes. Our understanding of fibrinolytic factors as simple fibrin and extracellular matrix (ECM) molecules that degrade enzymes has shifted. Fibrinolytic factors are also post-translational regulators of biomolecules, including chemo-/cytokines and cell surface receptors, and control the activity status of fellow proteases, such as matrix metalloproteinases (MMPs), ultimately establishing a proteolytic niche [1].

**Figure 1:**
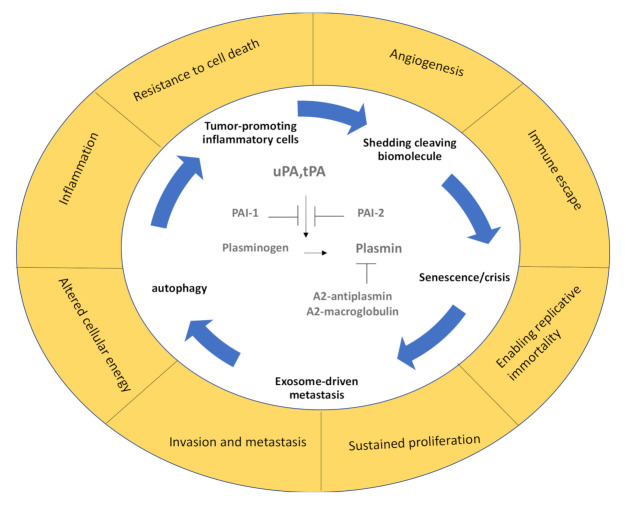
The fibrinolytic spiral during cancer. In the center, the main fibrinolytic factors and their endogenous inhibitors are depicted, which are surrounded by the pathogenic mechanisms they support. The outer circle summarizes the main hallmarks of cancer described by Hannahan and Weinberg [5]. PAI-1 and PAI-2: plasminogen activator inhibitor-1 and -2, tPA: tissue-type plasminogen activator, uPA: urokinase plasminogen activator.

Fibrinolysis is the process of blood clot dissolution following hemostasis and clot retraction, which is a process that keeps the blood flowing in vessels. The best-known fibrinolytic factor is the serine protease plasmin, which degrades fibrin (a major component of the clot) and degrades the basement membrane. Plasmin activity is controlled by alpha2-antiplasmin and alpha2-macroglobulin in the circulation. The plasminogen protein is an inactive zymogen that circulates in the blood plasma and is converted to the active protease plasmin by tissue-type plasminogen activator (tPA) and urokinase plasminogen activator (uPA). Plasmin activation also occurs after kallikrein and factor XII (Hageman factor) exposure, although with lower efficacies. The activity of uPA and tPA is inhibited by plasminogen activator inhibitor-1 and -2 (PAI-1 and PAI-2). PAI-1 can be produced by cancer cells [2], but also cancer-niche-associated cells, such as endothelial cells and activated platelets. An activated endothelium (due to thrombin, histamine, and bradykinin) and neurons or microglia release tPA. uPA is expressed on the bronchial epithelium, but also on some blood cells, such as monocytes, and in many cancer cells (Figure 1).

Cancer cells take advantage of the pericellular plasmin generation on cell surfaces. Depending on the stage of the disease and the receptors involved, fibrinolytic factors like plasmin(ogen), plasminogen activators (PAs), and their receptors promote or suppress cancer progression or inflammation (recently reviewed in [3]). uPA can bind to uPA receptor (uPAR), which is a glycoprotein that is linked to the plasma membrane by a glycosylphosphatidylinositol anchor. It is composed of three domains (D1–3). The general structure and function of uPAR have recently been summarized [4]. Other PA receptors include a-enolase, low-density lipoprotein receptor-related protein (LRP1), epidermal growth factor receptor (EGFR), annexin II, and certain integrins. Through their ability to interact with other membrane receptors, PA receptors influence intracellular signaling activation of tumor-associated pathways. 

Fibrinolytic factors control the critical hallmarks of cancer [5] (Figure 1). p53 is an important tumor suppressor that acts to restrict proliferation in response to DNA damage or the deregulation of mitogenic oncogenes by leading to the induction of various cell cycle checkpoints, apoptosis, or cellular senescence. Recent studies indicate that PAs and plasminogen receptors are p53 targets that accelerate primary tumor growth/metastasis and modulate anoikis (from the Greek word for “homelessness”) and senescence. Normal but not malignant cells undergo an apoptotic process termed “anoikis” after they lose contact with ECM or neighboring cells. Fibrinolytic factors, such as PAI-1 and PAI-2, can also be found in the senescence-associated secretory phenotype (SASP) complexes in cells that have reached a certain lifespan, promoting their entrance into the nondividing state of cellular senescence that can promote tumor growth.

Fibrinolytic factors also find their way into exosomes, which are secreted vesicles that harbor biomolecules, such as RNA, DNA, and glycans surrounded by a lipid bilayer (recently reviewed by [6]), and are known to enhance metastasis by establishing a prometastatic niche. From anoikis to senescence to autophagy and exosome-mediated metastasis promotion, along with other hallmarks of cancer [5], fibrinolytic factors modulate cancer progression. Below we review some recent studies in these exciting fields.

## 2. Plasminogen Contributes to Autophagy

Autophagy produces nutrients and energy to enhance cell survival through the breakdown of cytosolic components within the autophagosomes. Cancers use autophagy-mediated recycling (including the degradation of apoptotic mediators) to meet the metabolic demand for constant growth, proliferation, and cell survival. The native plasminogen is a single-chain glycoprotein, which contains the N-terminal peptide, five homologous Krinkle domains (K1–5), and the protease domain. Tumor-suppressive functions have been reported for individual domains or larger domain fragments (reviewed elsewhere [7]).

**Figure 2:**
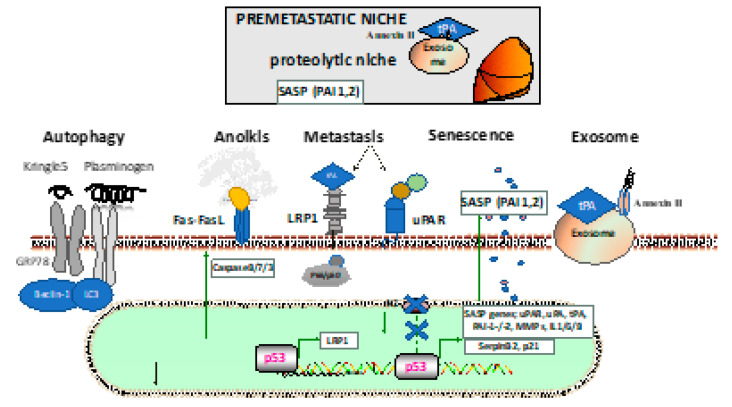
Establishment and interactions in the (pre)metastatic niche. The lower portion describes the mechanism through which fibrinolytic factors are involved in the cancer cells: autophagy through the Kringle 5 fragment or plasminogen; anoikis due to cell detachment with activation of the extrinsic apoptotic pathway through plasmin cleavage of the Fas ligand by plasmin; metastasis through pericellular plasmin activation via low-density lipoprotein receptor-related protein (LRP1) or uPA receptor (uPAR), alone or in complex with other membrane molecules (such as integrins or galectins); the participation of the senescence-associated secretory phenotype (SASP), including fibrinolytic factors like PAI-1 or PAI-2; the generation of fibrinolytic factor carrying exosomes that ensure the priming of the premetastatic niche in distant organs (such as the lung) with the establishment of the proteolytic niche. GRP78: glucose-regulated protein 78, LC3: microtubule-associated proteins 1A/1B light chain 3B, MMP: matrix metalloproteinase, PAI-1: plasminogen activator inhibitor-1, PAI-2: plasminogen activator inhibitor-2.

Treatment with the plasmin inhibitor tranexamic acid resulted in the formation of autophagosomes in B16-F1 cells with positive cellular staining for microtubule-associated proteins 1A/1B light chain 3B (LC3), which is the most widely used marker of the autophagosome membrane [7]. Interestingly, the plasminogen/plasmin effects on autophagy in cancer cells seem to depend on the cancer cell type. Tykhomyrov et al. showed that plasminogen-treated lung cancer cells upregulated beclin-1 (Atg6), which is important for the generation of the isolation membrane that engulfs cytoplasmic material to form the autophagosome, and caused cell detachment in cancer, but not normal cells (Figure 2). The prosurvival effects of plasminogen-boosted autophagy occurred after treatment with the full-length plasminogen, but not the Kringle 1-3 (K1–3) fragments of plasminogen [8].

The glucose-regulated protein 78 (GRP78) is an endoplasmic reticulum (ER)-resident chaperone that binds polypeptide chains noncovalently on the ER and then dissociates, facilitating proper protein folding and assembly and helping protein transport across the ER membrane. GRP78 plays an important part in maintaining protein stability, regulating protein folding, and inducing apoptosis and autophagy. Kringle 5 (K5) of the human plasminogen can bind to the 78 kDa GRP78 [8]. This protein is activated by caspases and evokes an autophagic response in endothelial cells. Endothelial cells exposed to K5 up-regulated beclin-1 levels and progressively increased the amount of antiapoptotic Bcl-2 complexed with beclin-1. Prolonged exposure to K5 ultimately led to apoptosis via mitochondrial membrane depolarization and caspase activation in endothelial cells. Knocking down beclin-1 levels decreased K5-induced autophagy but accelerated K5-induced apoptosis [8]. It seems that plasminogen-mediated survival signals depend on the cell type and certain parts of the plasminogen protein.

Fang et al. showed that K5 decreased the expression of GRP78 via the downregulation of phosphorylated ERK, leading to caspase-7 cleavage and tumor cell apoptosis [9]. Furthermore, K5 promoted the sumo/ubiquitin-mediated proteasomal degradation of hypoxia-inducible factor 1 alpha by upregulating von Hippel–Lindau protein, resulting in the reduction of vascular endothelial growth factor and thus suppression of tumor angiogenesis. 

## 3. Fibrinolytic Factors and Anoikis

The interstitial space between cells is filled with a matrix composed of type I, III, VI, VII, and XII collagens, proteoglycans, and glycoproteins, such as tenascin C and fibronectin, that are laid down by stromal cells (reviewed in [10]). Recent studies demonstrate that tumor tissues of various organs lay down specific matrisomes (ECM and ECM-associated proteins), including fibrinogen or plasminogen [7,8]. 

Cellular adhesion is mediated by integrin receptors and their ECM counterparts. The cellular loss of ECM contact induces programmed cell death, also referred to as anoikis, and maintains homeostasis within tissues. Cell death is often induced by the extrinsic death system, namely, the Fas–Fas ligand. In contrast, tumor cells need to be anoikis-resistant to metastasize. 

Earlier studies have demonstrated that plasmin generated from plasminogen on the cell surface (through tPA released from smooth muscle cells) induces cell retraction and fibronectin fragmentation, leading to detachment and morphological/biochemical changes that are characteristic of anoikis [11] (Figure 2). Likewise, uPAR–uPA dependent activation of ERK and PI3K/Akt through transcription of BCL2L1 contributes to anoikis resistance [12].

Plasminogen supports anoikis in normal cells. In the brain, cancer cells encounter reactive astrocytes that produce PA, leading to the production of plasmin, which induces carcinoma cell death through the production of the soluble Fas ligand from astrocytes and Fas-expressing cancer cells [13]. Under these conditions, anoikis occurs and metastasis is inhibited. To overcome this death sentence, tumor cells showed upregulated PAI-1 in the brain metastatic subpopulation of human lung adenocarcinoma cell lines and in the human mammary carcinoma cell lines, where this upregulation correlated with brain relapses in patients [13]. The upregulation of PAI-1 in cancer cells leading to plasmin inhibition was shown to prevent Fas–Fas-ligand-mediated tumor cell death, enabling the surviving cancer cells to metastasize.

## 4. PAI-2 and Senescence

The incidence of cancers increases with age. A key process in aging is senescence, where on a cellular level, cells lose their ability to proliferate, inhibit apoptosis, and change their metabolism and chromatin (reviewed recently by Wyld et al. [14]). Senescent cells secrete a plethora of factors, including proinflammatory cytokines and chemokines, growth modulators, angiogenic factors, and MMPs, which are collectively termed the SASP phenotype: interleukins (IL6, 7, 1, 1b, 13, 15); chemokines, including IL8; eotaxin; inflammatory cytokines, such as interferon-gamma; proteases, such as MMP1, 3, 10, 13, 14, TIMP1/2, PAI-1, PAI-2, tPA, uPA, and cathepsin B; receptors, such as uPAR; EGFR, FAS; insoluble factors, such as fibronectin, collagens, and laminin [15,16] (Figure 2). Physiologically, senescence serves as a tumor-suppressive mechanism that prevents the expansion of premalignant cells. However, the generation of SASP in chronically senescent cells seems to promote tumor progression.

The levels of PAI-2, which is produced from the *serpinb2* gene, were elevated in senescent human skin fibroblasts [17]. It was shown that PAI-2 is a direct downstream target of the tumor suppressor p53 following DNA damage. Not extracellular, but intracellular PAI-2 bound to and stabilized the cell cycle regulator p21 and mediated senescence. Kindlin-2 bound to p53 enhanced the expression of the senescence genes PAI-2 and p21 through binding to the PAI-2 and p21 promoters [18].

Decreased PAI-2 expression has been associated with increased tumor invasiveness and metastasis for several types of cancer. In 50% of PAI-2-deficient mice aged over 18 months, spontaneous malignancies of vascular origin were found [19]. Moreover, accelerated tumor growth was observed in PAI-2-deficient mice when injected with B16 melanoma or Lewis lung carcinoma cells. Chimeric bone marrow transplantation experiments established the nonhematopoietic compartment as the source of PAI-2 that augmented tumor growth in murine melanoma and lung carcinoma models.

## 5. Fibrinolytic Factors Help to Establish the Premetastatic Niche

Primary tumor growth and the metastatic process requires extensive crosstalk of integrin-carrying cells with the ECM. Anchoring the cell via integrins to the ECM, focal adhesion complexes connect the cell cytoskeletons to the ECM and sense the ECM conditions causing intracellular signaling and cellular behavioral responses. Defects in the focal adhesion complex involving components such as focal adhesion kinase (FAK) [20], SRC, and paxillin (PXN) promote cell transformation and metastasis. Nuclear PXN enhanced tumor angiogenesis by increasing tPA expression, resulting in LRP1-mediated NF-κB activation, a process involving the nonreceptor tyrosine kinase protein SRC [21]. 

The uPAR/LRP1/integrin complex that binds the beta-galactoside sugar-binding protein galectin-8 (gal-8) was shown to phosphorylate PXN and FAK, and activate JNK and the NF-κB pathway [22]. The importance of gal-8 in tumor growth was shown in gal-8 transgenic mice. Transgenic mice showed increased expression of proinflammatory cytokines (MCP1, IL1b/6, and TNF-α) and the prometastatic molecule RANKL [23]. Smaller tumors and a reduced number of lung metastases were found after orthotopic injection of E0771 cells into the fourth mammary gland in gal-8-knockout mice [22]. Previous studies demonstrated that membrane-associated gal-1 can serve as a tPA receptor on pancreatic cancer cells that increases tPA-mediated proteolytic activity and enhances ERK activation, cell proliferation, and the invasion of cancer cells and fibroblasts [24]. 

Inflammatory cells support tumor growth. Activation of NF-κB pathways, not only in tumor cells but also macrophages, is linked to the secretion of cytokines in the premetastatic niche. Fibrinolytic factors support angiogenesis and reprogram macrophages into M2 macrophages that express cytokines, chemokines, and proteases, promoting tumor angiogenesis, metastasis, and immunosuppression. Kubala et al. demonstrated that PAI-1’s LRP1-interacting domain regulates macrophage migration, while PAI-1’s C-terminal uPA-interacting domain induces M2 macrophage polarization through the activation of p38MAPK and NF-κB and the induction of an autocrine IL-6/STAT3 activation pathway [13]. DNA damage increased ⍺-enolase expression in mutant p53 isoform peripheral blood mononuclear cells (∆133p53, mΔpro (an isoform lacking the proline domain), and Δ122p53 (an isoform mimicking the human Δ133p53α p53 isoform)). When these cells were exposed to plasminogen, TNF-α expression increased, which could be blocked using a plasmin inhibitor. The tumors that developed in Δ122p53 mice had been reported to show increased proinflammatory features and were more aggressive than those developing in ∆133p53 mice [25].

Insulin-like growth factor binding protein-3 (IGFBP-3) is a p53 tumor-suppressor-regulated protein with two p53 binding sites in the first and second introns of the *IGFBP-3* gene (recent review by Cai et al. [26]). Cellular expression of IGFBP-3 is upregulated after treatment with growth inhibitors, such as anti-estrogens, TGF-β, retinoic acid, TNF-α, vitamin D, histone deacetylase inhibitor sodium butyrate, and anticancer dietary components (silibinin, apigenin, lycopene, resveratrol, curcumin, and quercetin).

IGFBP-3 ligands in the blood circulation include IGF-1, IGF-2, and the acid-labile subunit [27]. Earlier studies demonstrated that plasmin cleaves IGFBP-3 into fragments. The IGFBP-3 fragments have lower affinities for insulin-like growth factors (IGFs), resulting in the release of IGFs into target tissues, where they contribute to cell proliferation and metabolism [28]. IGFBP-3 can bind to the cellular receptors LRP1 [29] and transmembrane protein 219 [26]. LRP1 seems to be important for the cellular internalization of IGFBP-3. The IGFBP-3 and uPA mRNAs both increased in pancreatic ductal adenocarcinoma [30]. The expression levels of uPA, uPAR, IGF-1, and IGFBP-3 mRNA were significantly greater in pancreatic ductal adenocarcinoma than in benign mucinous cystadenomas’ control tissues [31].

The milk immunomodulatory glycoprotein lactoferrin binds and transports iron ions, and has antibacterial, antiviral, antiparasitic, and antiallergic functions, along with anticancer properties. Earlier studies in PC12 and N2a neuron-like cells demonstrated that lactoferrin functioned as an LRP1 signaling antagonist, inhibiting Trk receptor phosphorylation and ERK1/2 activation in response to enzymatically inactive tPA [32]. A recent study demonstrated that lactoferrin binds to plasminogen on the cell surface and blocks plasminogen activation by uPA [33]. The mutual binding sites of lactoferrin and plasminogen were mapped within the N-terminal region of lactoferrin, which was also encompassed in the bioactive peptide lactoferricin, and Kringle 5 of plasminogen, respectively. This study also demonstrated that lactoferrin administration blocked tumor cell invasion in vitro and plasminogen activation driven by *Borrelia*. These data indicate that plasmin controls tumor growth and invasion via ostensibly unrelated tumor-growth-modulating factors, such as IGFBP-3 or lactoferrin.

Tumor-growth-supporting cells include mesenchymal stem cells (MSCs). We found that tPA recruits MSCs into growing tumors [34]. The increased number of MSCs could be due to tPA’s effects on migration, but also might be due to its role in expanding MSCs, involving crosstalk between mesenchymal stem and endothelial cells [35,36].

Metastasis is responsible for about 90% of cancer-associated deaths. The development of metastases requires cancer cells to colonize at the metastatic site [37]. uPA and tPA receptors, such as uPAR, LRP1, integrins, and annexin II (AnxII), modulate intracellular signaling pathways, just like NF-κB, establishing a premetastatic niche [38,39], which is an environment prepared for tumor cell colonization in distant organ sites (Figure 2). 

LRP1 mediates its oncogenic effects through molecules such as uPAR or the chaperone hsp90-α. A role for LRP1 in pulmonary metastasis was established previously using CL16 cells (derivates of the human breast cancer cell line MDA-MB-435) in xenografted mice [40]. In murine models of melanoma, ApoE secreted by melanoma cancer cells suppressed tumor invasion and metastatic endothelial cell recruitment by binding to LRP1-positive melanoma cells and LRP8-positive endothelial cells [41]. Our group showed that the tPA-secreting B16 melanoma, but not niche cells, enhanced the tumor growth in wild-type, but not tPA-deficient mice. Furthermore, tPA, in part via LRP1, enhanced lung metastasis in the B16 melanoma model [34]. When LRP1 and tPA were restored in less aggressive, poorly metastatic melanoma cells, melanoma cell growth and lung metastasis were accelerated [34]. Tumor-derived exosomes are extracellular vesicles that carry and transfer molecules, such as proteins, lipids, microRNAs, and mRNAs [38], and these vesicles support tumor and distant premetastatic niche communication. Earlier studies demonstrated that tumor-derived exosomes contain the chaperone hsp90-α by releasing their cargo binds to tPA and extracellularly generating plasmin [42].

Annexin II, which is a receptor for both tPA and plasminogen (recently reviewed in cancer [12]) is highly expressed in exosomes derived from malignant cells, but less expressed in normal and premetastatic breast cancer cells [43]. Functional AnxII expression in exosomes (Exo-Anx II) was required for tPA-dependent angiogenesis, as neutralizing antibodies against tPA nullified the proangiogenic effects. Exosomes maintained the organ tropism. Maji et al. demonstrated that just as in the original cell lines themselves, exosomes of MDA-MB-831 caused brain metastasis, and exosomes of MDA-MB-4175 resulted in lung metastasis. Mechanistically, tumor-derived exosomes were found to colocalize with pro-cathepsin B and caused macrophage activation with IL6 and TNF-α secretion via stimulation of the p38, NF-κB, and STAT3 pathways [43]. Another interesting aspect of exosomes is that tumor-derived exosomes can confer an increased plasmin-generating capacity to a recipient cell in certain cell types [44] that might be important for ECM preparation in the premetastatic niche.

## 6. Plasminogen Receptors and Drug Resistance 

Myelosuppressive therapies, such as chemotherapy and irradiation, cause cell death and tumor regression, but also upregulate proteases, such as tPA/plasmin and MMPs [45,46]. An urgent and unsolved problem in cancer treatment is the occurrence of drug resistance. 

Plasminogen-binding receptors, such as uPAR [47], ⍺-enolase, and a low-dose-lipoprotein, such as LRP1 [48], have been proposed to mitigate resistance to cancer therapy.

The prosurvival signals of uPAR have been proposed to help cancer cells to escape the cytotoxic drug effects and contribute to tumor resistance (reviewed by Gonias and Hu [49]). Zhou et al. demonstrated that uPAR mRNA was increased in tumor exosomes of patients or cell line samples showing resistance to the epidermal growth factor receptor tyrosine kinase inhibitor geftinib when compared to geftinib-sensitive controls [50]. When uPAR was silenced or EGFR was knocked down, geftinib resistance could be overcome and tumor cell apoptosis occurred via the EGFR/p-AKT/survival signaling pathway. Similarly, the sensitivity to the BRAF inhibitor Vemurafenib in V600E mutant melanoma cells could be improved after uPAR silencing, leading to better tumor suppression [51]. Mechanistically, the authors demonstrated that a key to improved drug sensitivity was the disruption of the uPAR–integrin interaction.

As introduced above, senescent cancer cells can generate SASP factors that include PAI-1, PAI-2, tPA, and uPA, which could enhance tumorigenesis and establish an immunosuppressive environment. Therefore, the targeted removal of senescent cells (senolysis) is an emerging strategy in cancer treatments, particularly in combination with more traditional anticancer interventions. uPAR is expressed on senescent cells. Amor et al. demonstrated that uPAR-specific chimeric antigen receptor (CAR) T cells can efficiently ablate senescent cells, working as so-called senolytics (from the words senescence and -lytic, meaning “destroying”). They extended the survival of mice harboring lung adenocarcinoma that were treated with a senescence-inducing drug combination [52].

Chemotherapeutic drugs inflict DNA damage, which is sensed by p53, causing the activation of kinases that lead to a stable complex of p53 and its transcriptional target mdm, where the accumulation of p53 induces cell cycle arrest and apoptosis [14]. At low concentrations of DNA-damaging agents, p53 mainly activates prosurvival genes that try to repair the damage. At high concentrations of DNA-damaging agents, p53 activates proapoptotic genes. Chemotherapy is less effective in cancer cells as the tumor suppressor p53 is often mutated in cancer. Recent studies indicate that PA or plasminogen receptors are p53 targets. While LRP1 mRNA was upregulated following sublethal and lethal doses of irradiation (p53 stress), LRP1 protein was augmented only in response to sublethal stress [53]. It was shown that LRP1 translation was suppressed through miR-103 and miR-107, resulting in cell death [54]. As with uPAR, LRP1 mitigates prosurvival signals in tumor cells [55]. We recently showed that the chemotherapeutic drug bortozomib induced the expression of LRP1 and its ligand tPA in melanoma cells [34]. The chemosensitivity of melanoma cells could be improved when tPA or LRP1 was knocked down [34].

## 7. Conclusions

Here, we summarized recent studies showing the involvement of fibrinolytic factors, such as uPA, tPA, PAI-1, and PAI-2, and associated receptors, such as uPAR and LRP1, in modulating critical signaling pathways, and thereby contributing to the hallmarks of cancer. After their initial discovery as enzymes involved in clot dissolution, fibrinolytic factors and their ever-increasing coterie of known binding partners, including cellular receptors, enables them to contribute to every hallmark of cancer. We reviewed data on how they can (1) resist cell death through anoikis, (2) drive angiogenesis through M2 macrophage programming, (3) enable replicative immortality through the suppression of autophagy, (4) activate invasion and metastasis through exosomes, (5) evade growth suppressors, and (6) sustain proliferative signals/growth [5]. Their presence is required to establish a proper premetastatic niche.

Fibrinolytic factors and, here, mainly their receptors, such as uPAR, are important accomplices in enabling cancer cells to flourish within their primary or premetastatic lesions and to escape conventional antitumor therapy. The exploitation of fibrinolytic factor-associated receptors uPAR or LRP1 as novel drug targets in combination with conventional therapy is a growing field of research undergoing translation into the clinic.
